# The complete mitochondrial genome sequence of *Bryopsis plumosa*

**DOI:** 10.1080/23802359.2020.1721031

**Published:** 2020-02-06

**Authors:** Hongbin Han, Yan Li, Song Wei, Zongling Wang, Xuelei Zhang

**Affiliations:** a First Institute of Oceanography, The Ministry of Natural Resources, Qingdao, China;; b Laboratory of Marine Ecology and Environmental Science, Pilot National Laboratory for Marine Science and Technology (Qingdao), Qingdao, China;; c Laboratory of Marine Ecology and Environmental Science, Qingdao National Laboratory for Marine Science and Technology, Qingdao, China

**Keywords:** Macroalgae blooms, mitochondrial genome, *Bryopsis plumosa*, phylogenetic analysis

## Abstract

In 2015, a novel macroalgae began to bloom in Qinhuangdao on the coast of the Bohai Sea, Northern China. *Bryopsis plumosa,* a green macroalga, is one of the causal species of the macroalgae blooms, which have severely affected the environment and ecosystem on the coast of Qinhuangdao. In the present study, we sequenced the mitochondrial genome of *B. plumosa* for the first time (GenBank accession number MN853874). It was found that the ring-shaped genome was made up of 110,912 base pairs, including 29 protein-coding genes, 23 tRNAs, and 2 rRNAs. Phylogenetic analysis showed *B. plumosa* had close genetic relationships with algae *Ulva prolifera, Ulva linza*, and *Ulva flexuosa.* The present data will provide important information on the phylogenetics and molecular evolution of *Bryopsis* species.

Macroalgae blooms are caused primarily by the excessive growth and accumulation of macroalgae (Schories and Reise 1993). In China, the first serious macroalgae blooms were caused by *Ulva prolifera* and have occurred annually from 2007 to 2019 along the coast of the Yellow Sea, China, seriously affecting marine environment and ecological services functions (Liu et al. 2009, 2010, 2013; Xiao et al. 2013). Macroalgae blooms are likely to reoccur in other sea areas when exposed to appropriate oceanographic conditions, which is a matter of great concern to government officials and scientists in China (Liu et al. 2009). In 2015, another macroalgae bloom occurred in the coastal area in Qinhuangdao City of Hebei Province on the western coast of the Bohai Sea, and it has continued to recur every spring to summer since then (Song et al. 2019a). *Bryopsis plumosa* is one of the causal species of the macroalgal blooms and it is widely distributed along the coasts of the Yellow and Bohai Seas. (Song et al. 2019b). mtDNA has been used as molecular markers and has played important roles in genetic structure, species identification and phyletic evolution (Melton et al. 2015). Therefore, in this study, we determined the complete mitogenome sequence of *B. plumosa*.

*Bryopsis plumosa* was collected from the Qinhunangdao coastal area of the Hebei Province (39°49′54.69″N, 119°31′30.50″E). The specimens were preserved at the Marine Ecology Research Center of the First Institute Oceanography, Ministry of Natural Resources in Qingdao (Accession number: YZ06). The shape of the genome of *B. plumosa* is annular with GenBank accession number MN853874. The content of A + T is 63.74%. The complete mitochondrial genome sequence is 110,912 bps. There are 29 protein-coding genes in the genome, including 9 *rps* genes, 8 *nad* genes, 5 *atp* genes, 3 *rpl* genes, 3 *cox* genes and 1 *cob* gene. In addition, 23 tRNAs and 2 rRNAs (*rrl* and *rrs*) are non-coding genes included in the genome. All of the coding genes begin with ATG except for *psbC*, which begins with AAT. *nad*7, *nad*2, *rpl*5, *rps*13, *cob*, *nad*4L, *rps*11 and *rps*4 all terminate with TAG while the other 21 genes terminate with TAA.

To determine the phylogenetic position of *B. plumosa*, 17 complete mitochondrial genome sequences were obtained from the Genebank database. The phylogenetic tree of *Bryopsis plumosa* and other 17 species was constructed based on core genes. The single-copy homologous genes (core gene) were identified by common/unique gene analysis and then those core genes were aligned using MUSCLE v3.8.31 software. The maximum likelihood (ML) methods were performed for the phylogenetic analysis using PhyML 3.0, and the bootstrap was 1000. The results showed that *B. plumosa* is the closest sister species of *Ulva prolifera, Ulva linza*, and *Ulva flexuosa* (Figure 1). Therefore, we conclude that the complete mtDNA genome sequence obtained in this study will be useful for studying the phylogenetic history of *B. plumosa* and its related species.

**Figure 1.**
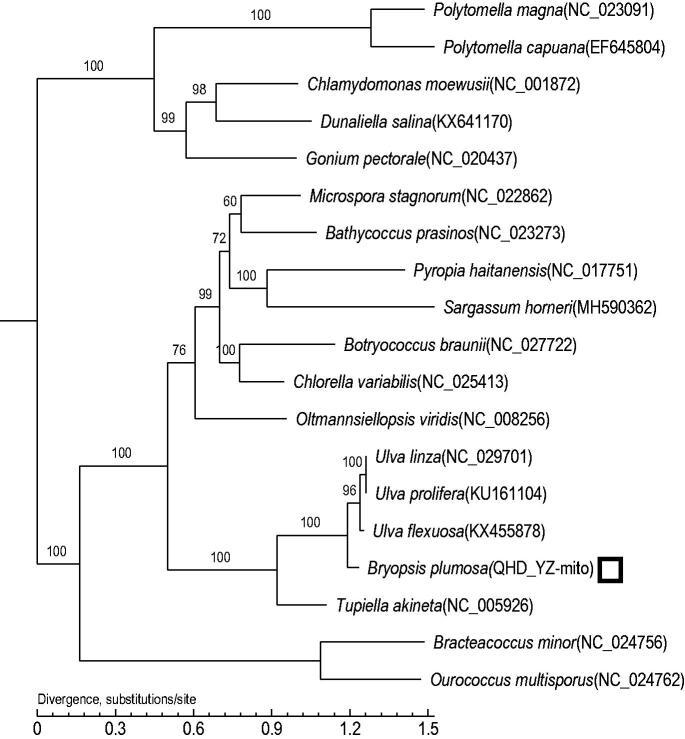
Maximum-likelihood (ML) tree based on the complete chloroplast genome sequences of 7 species. The numbers on the branches are bootstrap values.
